# Analysis of Actual Fitness Supplement Consumption among Health and Fitness Enthusiasts

**DOI:** 10.3390/foods13091424

**Published:** 2024-05-06

**Authors:** Paolo Renzo Espeño, Ardvin Kester S. Ong, Josephine D. German, Ma. Janice J. Gumasing, Ethan S. Casas

**Affiliations:** 1 School of Industrial Engineering and Engineering Management, Mapúa University, 658 Muralla St., Intramuros, Manila 1002, Philippines; 2 E.T. Yuchengo School of Business, Mapúa University, 1191 Pablo Ocampo Sr. Ext., Makati 1204, Philippines; 3 Department of Industrial and Systems Engineering, Gokongwei College of Engineering, De La Salle University, 2401 Taft Ave., Manila 1007, Philippines

**Keywords:** fitness supplements, health belief model, theory of effort of minimization, theory of planned behavior, structural equation modelling

## Abstract

With the rising popularity of fitness supplements, understanding the factors driving their consumption behaviors is crucial. This study investigated the actual consumption of fitness supplements utilizing the Theory of Planned Behavior (TPB), Health Belief Model (HBM), and the Theory of Effort Minimization in Physical Activity (TEMPA) frameworks. The TPB provided insights into how attitudes, subjective norms, and perceived behavioral control influence individuals’ intentions to consume fitness supplements. Additionally, the HBM sheds light on perceived effectiveness, benefits, barriers, and health motivation related to supplement consumption. Integrating the TEMPA framework further investigated the role of effort minimization in physical activity contexts. Through an online questionnaire, data were collected from a sample of 250 fitness supplement-consuming respondents. SEM analysis revealed significant associations between behavioral intentions and attitudes, perceived behavioral control, health motivation, and controlled precursors. However, it was seen that subjective norms, perceived effectiveness, perceived benefits, and automatic precursors were deemed insignificant. The findings contribute to a comprehensive understanding of the factors shaping actual consumption behaviors, offering valuable implications for marketers, health professionals, and policymakers seeking to promote informed and responsible supplement use among health and fitness enthusiasts.

## 1. Introduction

Supplementary drinks or fitness supplements are commonly used to enhance an individual’s performance in various sports and health settings, catering to both competitive and non-competitive athletes [1]. One widely known supplement for athletes and fitness enthusiasts is protein supplements like protein shakes. Others may be whey protein, caffeine, and creatine, to name a few. These supplements play a crucial role in helping individuals increase muscle mass, recover faster, and enhance their overall performance [2]. Whey protein stands out as a top-quality protein source, containing more essential amino acids than traditional protein sources. Its numerous benefits have made it a popular choice for snacks and drinks among consumers [3]. Another widely embraced supplement is caffeine, which is found in many sports and food supplements. Caffeine reduces perceived effort, minimizes fatigue and pain, and proves to be effective for endurance and high-intensity activities, which is the choice of consumers [4].

Creatine monohydrate is another well-known supplement used to gain muscle mass and support performance and recovery. It is known not to increase fat mass and remains effective even when taken in recommended doses [5]. Despite its popularity in the fitness and sports industry, evidence suggests that creatine can benefit not only athletes but also the elderly and the general population [6]. Branched-chain amino acids (BCAA) also offer a plethora of benefits for consumers. As explained by Sanz et al. [7], BCAAs are stored directly in muscles and serve as the raw materials needed to build new muscle. This contributes to the overall process of strengthening muscles and alleviating post-workout soreness. Consumers often integrate these supplements into their routines with the aim of optimizing the outcomes they wish to achieve and support overall well-being [1].

The sports supplement industry makes for an impressive market share of nearly $12 billion in the health/wellness portfolio and is projected to be worth $24.4 billion by 2025. In a study by Burke [4], which investigated 20 published studies about the prevalence, methods, and reasons for supplement consumption, it is evident from the findings that supplement consumption is prevalent among athletes and even more so in individuals competing at higher levels. It can also be confirmed that most gym-goers generally use dietary supplements, with a prevalence exceeding 40%, with a prevalence of 44% in Portugal and 81% in South Africa [8]. In a set of different studies, the prevalence among gym-goers ranged from 29.4% to 47.5%, and these findings remained consistent. It was also seen that among 55 Spanish basketball players and English elite track and field athletes, the prevalence was 58% and 86%, respectively [9].

When investigating the dietary habits of combat sports athletes in the Philippines, the findings indicated that the overall knowledge of the respondents on nutrition and dietary habits was exceptional. However, the understanding of individuals about the excess intake of vitamin and mineral supplements was poor [10]. Similarly, Elsahoryi et al. [11] assessed the trends on dietary supplements among students and found that supplements were deemed essential; however, their knowledge of them was concluded to be insufficient. The findings of these studies implicated a need for educational intervention on supplements. Furthermore, the presence of unlicensed and unsafe supplements poses a threat to uneducated consumers. The country’s Food and Drug Administration (FDA) issued a strong public caution regarding the use of 11 dietary supplements in 2019. This was because the products have not undergone the regular evaluation process of the agency, making it impossible to ensure the safety and quality of the items [12]. Additionally, the availability of dietary supplements that claimed to help in weight loss has increased in drugstores and supermarkets. Many of these supplements contain various ingredients, typically herbs, vitamins, and minerals, caffeine, and laxatives, that when taken collectively, have unclear effects [13].

Individuals leading luxurious lifestyles tend to exhibit a higher inclination toward the use of supplements [14], indicating a correlation between lifestyle and the tendency to use supplements. Nagar [15] found attitude to be the most influencing predictor of consumers’ consumption intention of gym supplements, implying that customers’ attitudes can be affected by risk and benefits, social influence, and health consciousness. On the other hand, Campbell et al. [16] highlighted perceived behavioral control as not highly influenced by attitude and social norms. In another study, additional information on labels was found to significantly impact consumers’ behavioral intention to use dietary supplements [17]. The study further explained that consumers often consider using the supplement if its risks and benefits are well communicated on the label. It was also reported that customer consumption behavior with regard to fitness supplements is affected by their comprehension of the side effects [18]. Moreover, it was indicated that knowledge is a significant variable in consumers’ attitudes, beliefs, and intentions toward the use of supplements [19]. As knowledge grows, parallel improvement in understanding, belief, and intention to use supplements is present.

Jairoun et al. [20] suggested a positive perception regarding the use of fitness supplements. That is, the consumption of fitness supplements has been perceived to lead to doping, and users demonstrate stronger intentions and attitudes toward it [21]. These studies highlighted the gap in proper education regarding supplements that can distort individuals’ ability to make informed and responsible choices about their consumption, potentially putting their health at risk [22,23]. Moreover, health behavior among consumers was barely addressed among the studies. It could be posited that scarce to no studies have been conducted regarding fitness supplement consumption, especially in the Philippines, where gym-goers and fitness enthusiasts are abundant [24]. The emphasis Filipinos place on both wellness and aesthetics has fueled a rise in gym participation nationwide. Consequently, there has been a notable uptrend in the demand for nutraceuticals, such as protein bars and powders, despite their higher costs [25].

This study aimed to examine factors affecting the actual consumption of fitness supplements among health and fitness enthusiasts. This study employed a theoretical framework that combined the Theory of Planned Behavior (TPB), the Health Belief Model (HBM), and the recently developed Theoretical Framework of Motivation to Pursue Exercise Activities (TEMPA). By integrating these frameworks, this study provided a more holistic understanding of the psychological and motivational factors that contribute to the consumption behavior and intention of fitness supplements. The significance of this study lies in its potential to contribute insights to both academia and the health and fitness industry. Understanding the determinants of supplement consumption can aid in developing targeted interventions, educational programs, and marketing strategies to promote the responsible and informed use of fitness supplements among enthusiasts, as well as enhancing overall health and well-being. Furthermore, the incorporation of the framework represents a novel approach to studying health-related behavior, offering a more nuanced perspective on the intricate interplay of psychological, belief-based, and motivational factors in the context of fitness supplement consumption, which could be applied and extended among other related studies.

## 2. Conceptual Analysis and Hypotheses Build-Up

**Figure 1:**
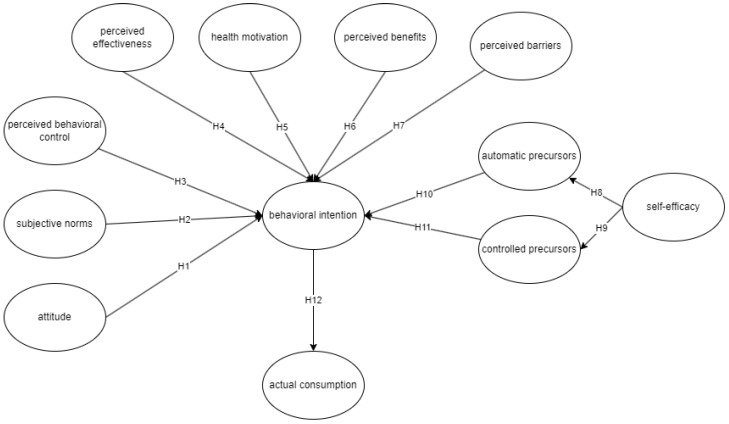
Conceptual framework combining TEMPA, HBM, and TPB to assess behavioral intention and actual consumption of fitness supplements.

Figure 1 presents the conceptual framework of this study, integrating TPB, HBM, and TEMPA. A total of 12 hypotheses were generated along with 12 latent variables. Among the theories, the TPB is said to be a frequently used behavioral model that aids in investigating shifts in people’s behavior. This model operates on the premise that behavior is intentional, enabling the prediction of actions [26]. On the other hand, the HBM is a well-researched framework for understanding health behavior. It seeks to anticipate behavior related to health by examining specific patterns of beliefs [27]. Lastly, several studies have employed TEMPA to align with individuals’ physical efforts. TEMPA is a comprehensive model assessing individuals’ tendency to engage in physical activities, incorporating factors that may promote their intentions [28].

Dealing with TPB domains, several studies have justified the effects of behavioral intention on up-taking dietary supplements. In this study, attitude refers to a disposition to react positively or negatively to a behavior [29]. It is evident in multiple studies that attitude is a variable that has a significant impact on consumer’s behavioral intention. In context, Conner et al. [30] investigated the environmental influences that contributed to the decision of women to use dietary supplements. The results showed that stronger intentions to use supplements were associated with positive attitudes. Liu et al. [31] assessed the effect of the fear of COVID-19 on purchase behavior on dietary supplements. Their findings supported that attitude, which, alongside other behavioral parameters, significantly affected purchase intention. Moreover, Nagar [15] examined the choice of gym supplements that gym-goers would purchase. The findings revealed that gym-goers’ intention to buy supplements is influenced by their attitude, which is shaped by perceived risk and benefit, social influence, and health consciousness.

On the other hand, subjective norms represent an individual’s perception of others’ opinions regarding the execution of a particular behavior, affecting the intentions of Iranian adolescent girls to intake iron and vitamin D supplements [32]. While Housman [33] analyzed the usage of sports-related supplements of elite female athletes, it was noted that their perceptions were influenced greatly by the beliefs held by significant people around them. Participants expressed that the strongest influences on their behavior came from parents and teammates. The findings of Haubenstricker [34] also stated that subjective norms and attitude were the most influential drivers in female bodybuilders’ intentions to use dietary supplements.

In addition, Lino et al. [35] conducted a study to investigate the attitudes and beliefs on dietary supplements among HIV-positive black women. The findings of the study indicated that perceived behavioral control is a significant predictor of intention to use dietary supplements. Similarly, Chen et al. [36] examined the effects of the perceived benefits of vitamin D supplementation intention. The results presented that the levels of intention to take vitamin D supplements were accurately predicted by factors under the TPB domains; the highest was perceived behavioral control. Pawlak et al. [37] investigated the drivers of multivitamin consumption in female Caucasian college students. Perceived behavioral control, along with attitude, were found to have a significant impact on their behavioral intentions. Thus, in this study hypothesized the following:

**H1.**  *Attitude has a significant direct effect on behavioral intention.*

**H2.**  *Subjective norms have a significant direct effect on behavioral intention.*

**H3.**  *Perceived behavioral control has a significant direct effect on behavioral intention.*

For health and consumers’ belief, Hanna and Hughes [38] investigated the perspective of the public regarding decision-making regarding over-the-counter drugs and their stance on the evidence supporting its effectiveness. The results of the study showed that the perceived effectiveness of the medicine influenced their intention to purchase. Similarly, Münstedt et al. [39] investigated the perceived effectiveness of complementary and alternative medicine. The findings indicated that belief in the effectiveness of complementary and alternative medicine were motivating factors. Increasing perceived effectiveness results in a positive attitude and stronger purchase intention, underscoring the importance of the factor in consumer behavior [40]. Hence, the following was hypothesized:

**H4.**  *Perceived effectiveness has a significant direct effect on behavioral intention.*

Motivation for health involves an individual’s readiness to modify their behavior for the sake of enhancing their well-being [41]. In the study by Ataei et al. [42], it was explained that a significant influence of health motivation, among other factors, on farmers’ intentions to adopt green and safer pesticides was seen after they were aware of the benefits of utilizing it. This signified that attitude toward health and the prevention of diseases is one of the main drivers to utilize the greener alternatives. Moreover, Sirico [43] observed that Italian students often consumed food supplements with a focus on overall well-being. They underscored that the respondents’ pursuit of maintaining good health is a priority. Likewise, Kovács [44] found that leisure-time athletes primarily took supplements as a proactive measure to maintain their health. These findings collectively highlighted the trend of individuals engaging in behaviors aimed at improving their health. Therefore, the following could be hypothesized:

**H5.**  *Health motivation has a significant direct effect on behavioral intention.*

Yazdanpanah et al. [45] found that the key factor influencing the intention to consume organic food among Iranian young adults is the perceived health benefit. This suggests that individuals are inclined to choose organic foods due to their perceived health benefits for both themselves and the environment, thereby increasing the likelihood of choosing organic options. In a similar vein, Saghafi-Asi [46] identified perceived benefits as a robust predictor of weight management behavior, and Shitu [47] reported a positive correlation between perceived benefits and preventive behavior against COVID-19. Thus, this study hypothesized the following:

**H6.**  *Perceived benefit has a significant direct effect on behavioral intention.*

Perceived barriers, on the other hand, are the beliefs that carrying out a behavior is restricted due to psychosocial, physical, or financial factors. When assessing the predictors of the intention to receive the COVID-19 vaccine, low perceived barriers were an important predictor of a definite intention to take the vaccine [48]. Bhandari [49] found that perceived barriers negatively affected the safety behaviors of farmers to use safer pesticides and adhere to safety behaviors; the findings indicated that educational programs and training were advised to mitigate the effect of these perceived barriers. Similarly, Pinho et al. [50] found that perceived barriers also had a significant effect on healthy eating. This suggests that individuals’ decision to carry out behaviors that positively affect health are significantly affected by perceived barriers. To which, this study hypothesized the following:

**H7.**  *Perceived barriers have a significant direct effect on behavioral intention.*

Self-efficacy is one’s belief in being able to succeed in any task that they are given. This trait can be general or specific, allowing individuals to have a range of self-efficacy about themselves [51]. Upon investigating the relationship between dietary supplements and the general self-efficacy levels of athletes, the findings showed that athletes with lower self-efficacy tend to consume more supplements [52]. Broelz et al. [53] found that giving athletes a supplement with high salience caused them to work harder than products with low salience, underscoring the psychological benefit of supplements. Another study focused on ergogenic supplements’ effects on the performance of cycling athletes; they had a positive effect on the speed and endurance of the cyclists [54]. Similarly, López-Torres et al. [55] discovered that the consumption of ergogenic supplements aided in the performance of female athletes and enhanced their efficacy to reach specific physical needs and goals. In addition, Gacek [56] investigated the determinants of nutritional choices of Polish handball players; it was found that self-efficacy and the consumption of vegetables are positively correlated, thus indicating their belief in achieving specific goals. Encompassing those associated with both health and sports, it underscores the importance of a rational nutritional model. Thus, the following was hypothesized:

**H8.**  *Self-efficacy significantly affects automatic precursors.*

**H9.**  *Self-efficacy significantly affects controlled precursors.*

For the precursors, TEMPA explains why individuals aspiring to engage in physical activity may not do so [57]. The framework considers both the automatic reactions triggered by physical activity cues and the inherent desire to avoid exerting too much physical effort. This explains that a person’s movements are swayed by automatic and controlled precursors. These precursors are triggered by the cues related to the movement, and how individuals feel about these cues was also said to affect the perceived effort that is needed to perform the movement [58]. For these precursors to be effective and drive an individual to engage in physical activity, the precursors supporting the intention should outweigh the negative precursors that reduce effort [28]. Ong et al. [24] exhibited that health and well-being are preceded by a person’s self-efficacy and its increase, which also increases their behavioral intention. Thus, the following was theorized:

**H10.**  *Automatic precursors have a significant direct effect on behavioral intention.*

**H11.**  *Controlled precursors have a significant direct effect on behavioral intention.*

Goulet et al. [59] investigated the predictors of the use of performance-enhancing drugs (PED) among young athletes. To which, their study presented that all behavioral factors had a positive influence on behavioral intention. The results of the study suggested that psychosocial factors had a significant effect on their use of PEDs. In another study, Samoggia and Rezzaghi [60] investigated the effect of the consumption of products that contain caffeine on sports performance. Behavioral intention was the dependent variable in the study, presenting a positive outcome from the consumption of the products. Furthermore, in a study investigating the role of education on the use of energy supplements, Mehri et al. [61] found that behavioral intention had the most significant impact. Educational interventions lowered the behavioral intention of the participants, which ultimately decreased the intake of the supplements. Relating to the positive results of studies, the study hypothesized the following:

**H12.**  *Behavioral intention has a significant direct effect on the actual consumption of fitness supplements.*

## 3. Methodology

### Participants

The questionnaire was voluntarily answered by 250 respondents and was collected via a purposive sampling technique. The data collection period started in November 2023 and finished in February 2024, and respondents were composed of individuals residing in the Philippines who consume any kind of fitness supplement. The responses were gathered through Google Forms and were answered in face-to-face interviews among gym-goers.

Table 1 shows the descriptive statistics of the demographic profiles of the respondents. A total of 57.6% are male, while 42.4% are female. The respondents’ age ranges were 15–24 (74.8%), 25–34 (20.4%), 35–44 (3.6%), 45–54 (0.8%), and 55–64 years old (0.4%). The respondents’ educational status consists of 1.60% junior high school graduates, 55.2% senior high school graduates, 2.8% vocational graduates, 39.6% college graduates, and 0.8% master’s degree graduates. Moreover, the respondents’ employment status was asked, and it showed that 70% are students, 2.4% are unemployed, and 27.6% are employed/self-employed.

The survey instrument (Appendix A) encompasses five aspects of respondents’ perspectives toward fitness supplement consumption. Initially, Table 1 presents the respondents’ demographic profile. Consequently, the Theory of Planned Behavior (TPB) framework consisting of three latent variables assessed the respondents’ attitude, subjective norms, and perceived behavioral control toward their consumption of fitness supplements. Followed by this, the Health Belief Model (HBM) framework consisting of four latent variables assessed the perceived effectiveness, perceived benefits, perceived barriers, and health motivation of the respondents. The Theory of Effort Minimization in Physical Activity (TEMPA) framework involves another three latent variables, including self-efficacy, automatic precursors, and controlled precursors. The final section of the questionnaire gauged the behavioral intention and actual consumption patterns of the respondents. In totality, the questionnaire comprises 12 latent variables, utilizing a 5-point Likert scale ranging from 1 as “Strongly Disagree” to 5 as “Strongly Agree”.

To measure factors affecting actual consumption, the Structural Equation Modeling (SEM) was applied in this study using SMART PLS v3.0. According to Ampofo and Aidoo [62], SEM is a prominently used multivariate tool for assessing the direct and indirect relationships between latent variables. The theory simplifies the relationships by constructing a path model to depict the effects resulting from the latent variables. Wathanakom [63] utilized SEM to assess the factors influencing Generation Y consumers’ vitamin and nutritional supplement purchasing intentions. The outcome of the study outlined the direct and indirect effects of the various factors, and their approach was said to be valuable to design campaigns to increase awareness about the significance of vitamins and nutritional supplements, aiding consumers in selecting the suitable supplements for purchase. In addition, Nystrand and Olsen [64] investigated the intention of consumption toward functional foods with an extended TPB model; their results enhanced insight into consumer motivation for functional food consumption and suggested potential benefits for the food industry. Moreover, the SEM results supported hypotheses related to predictors of intention and consumption frequency for functional foods, highlighting the importance of self-efficacy, attitudes, social norms, and control beliefs in consumers’ decision-making processes.

## 4. Results

**Figure 2:**
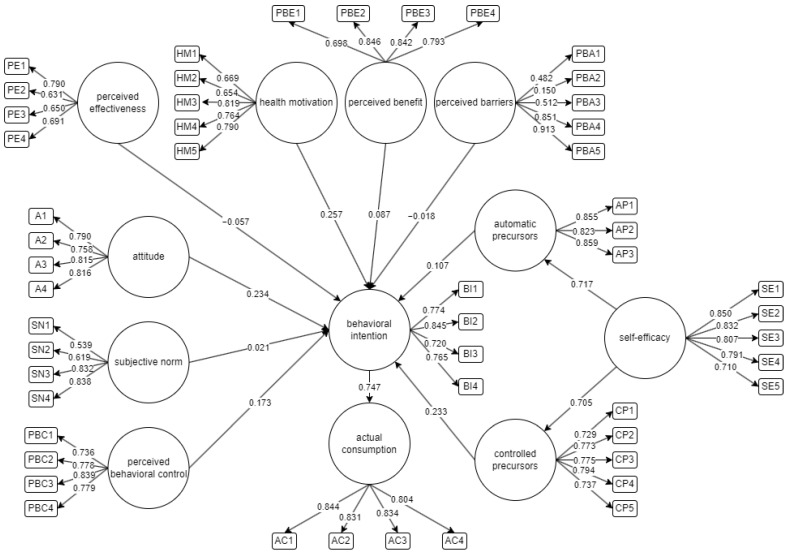
Initial Structural Equation Model to assess behavioral intention and actual consumption of fitness supplements.

The initial structural equation model (SEM) illustrating the factors influencing the intention to consume fitness supplements is represented in Figure 2. The indicators of latent variables serve as measures to gauge the validity of the relationship between observed data and the underlying construct. The survey questionnaire allows respondents to self-evaluate the significance of factors that affect their behavioral intention to consume fitness supplements. This model indicates whether an indicator affects and influences an individual’s intention.

**Figure 3:**
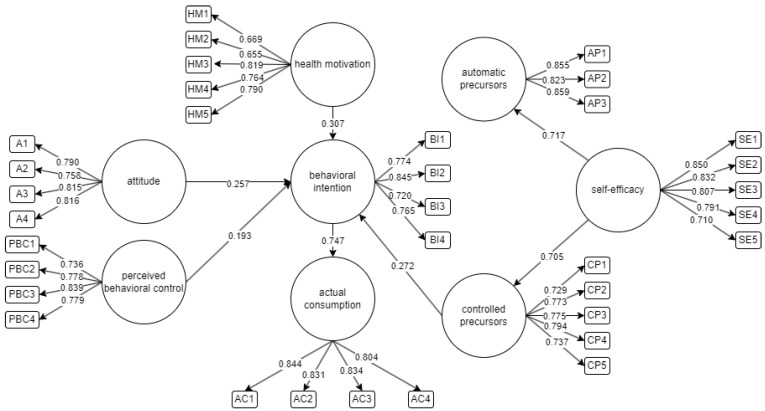
Final structural equation model.

Table 2 displays the reliability and validity values for the final model. Not all factor loadings sufficiently capture the latent variability. Therefore, items with initial loading values that were less than 0.7 were extracted from the final loading. Chronbach’s apla (α), composite reliability (CR), and the average variance extracted (AVE) are then used to measure the internal consistency, reliability, and validity. Additionally, the cutoff value for the convergent validity of the AVE should be higher than 0.5. All values made the cutoff, which indicates consistency and reliability across the test item sample. This implies that each construct from this model may be classified as valid and reliable. Presented in Figure 3 is the final SEM for this study.

In Table 3, the discriminant validity includes both the Fornell–Larcker criterion and the heterotrait–monotrait ratio. It illustrates the correlation between each latent variable and evaluates the structural model [65]. As suggested, the threshold value for the HTMT ratio should be less than 1 (<1) indicating a correlation between two latent variables. However, if the ratio is greater than 1, a lack of discriminant validity is evident. Hence, the authors recommend a HTMT ratio lower than 0.85 or 0.90 for constructs to avoid overlap. As shown in Table 4, the HTMT ratio fell within the desired threshold.

Table 4 shows the model fit indices conducted to illustrate the proposed model’s reliability. The table indicates that all parameter estimates met the suggested cutoff, representing the suitability of the suggested model. The values of d_ULS and d_G were 1.205 and 5.316, respectively. With this, the results reflected excellent batch between the data and measurement model, indicating that the model’s quality is suitable to explain the data (Table 5).

## 5. Discussion

Fitness supplements are products designed to complement a person’s diet and exercise routine, with the aim of enhancing various aspects of health, fitness, or performance. The purpose of this study was to investigate the factors that influence the consumption of fitness supplements. A total of 250 individuals answered the online questionnaire, which accounted for 12 latent variables that were integrated from the Theory of Planned Behavior (TPB), the Health Belief Motivation (HBM) framework, and the Theory of Effort Minimization in Physical Activity (TEMPA) framework.

Attitude was seen to have a significant effect on behavioral intention to consume fitness supplements (β: 0.257; *p* < 0.001). Based on the established constructs, consumers find spending money on fitness supplements worthwhile and useful. This implies that consumers benefit from investing in fitness supplements and emphasize the importance and value of them. This is similar to the findings in the study by Liu [34], which highlighted attitude as a significant factor affecting the intention to consume supplements because consumers see purchasing supplements as a sensible and helpful choice for them. In another study, Wathanakom [66] showed that individuals who perceive gym supplements positively or associate them with health benefits or improved fitness are more inclined to consider using them.

Subjective norms were observed to have an insignificant impact on the behavioral intention to consume fitness supplements (β: 0.021; *p* = 0.688). This indicates that the decision of individuals to purchase and consume fitness supplements is a choice based on their own will and does not adhere to, nor is it influenced by, the standards or insights of other individuals, whether it be people important to them or celebrities who endorse the supplements. This result is aligned with the findings of Kitcharoen and Vongurai [69], whose findings indicate that subjective norms did not have any direct influence on behavioral intention to consume dietary supplements. It underscored how international companies appoint social media influencers as their brand ambassadors in the hopes that these brand ambassadors will foster a positive outlook on dietary supplements, supposedly increasing an individual’s inclination to use dietary supplements. However, it contrasts with the studies by Alami [32], which underscored that the influence of friends, parents, and teachers was significant in dietary behavior toward iron and vitamin D, as they served as role models and provided social support. Moreover, Haubenstricker et al. [34], found that bodybuilders in in-season competitions are influenced by those around them, including trainers, workout partners, and social media influencers.

Meanwhile, perceived behavioral control significantly affected behavioral intention, as well (β: 0.193; *p* = 0.004). Consumers exhibit a sense of control and ease over the consumption of fitness supplements, viewing it as manageable and straightforward. Moreover, this indicates the voluntary nature of this choice, highlighting the individual’s sense of control and decision-making. This is consistent with the findings of Chen et al. [36], which found that perceived behavioral control was a significant driver in the intention to consume vitamin D supplements, because individuals are more likely to engage in the consumption of supplements if they find it easy to perform, and that past experiences with supplementation shapes perceived behavioral control, where positive experiences boost confidence and negative ones create doubts. Additionally, Lino [35] also found that perceived behavioral control had a significant impact on the intention to use dietary supplements; the findings indicated that individuals with a greater sense of control over the use of dietary supplements demonstrated stronger intentions to use them. This suggests that individuals who felt more in control of their treatment were more likely to express positive beliefs and intentions regarding the use of dietary supplements. This is contrary to the findings of Al-Swidi et al. [70], whose results indicated that perceived behavioral control had no significant effect on the purchase intention of organic foods; the lack of significance of perceived behavioral control in predicting organic food buying intentions in this study is attributed to a combination of cultural factors, dependence on subjective norms, and the emerging nature of the concept in the studied population.

In this study, perceived effectiveness was found to have an insignificant effect on behavioral intention (β: −0.057; *p* = 0.429). Based on the constructs, this indicates that consumers do not deem fitness supplements as mandatory. The responses implied a frail view regarding the importance of using supplements for well-being, energy levels, and daily performance. These findings are similar to Burke and Manore [71], which stated that individuals whose diets provide adequate energy and nutrients can support exercise performance and maintain nutritional intake without the need for supplements. Furthermore, Jordan et al. [72] stated that micronutrient supplements are not mandatory for athletes who consume nutrient-dense food that provide high levels of energy, further highlighting the optionality of supplements. These results contrast those of Bussicott et al. [73], who found that perceived effectiveness played a significant effect in shaping the usage patterns of complementary medicine (CM) products. The reliance on information from family and friends, coupled with the occurrence of adverse effects in more than half of the participants, underscores the impact of perceived effectiveness on consumer choices. Despite potential risks and limited clinical evidence, CM weight-loss products were perceived as accessible, safe, and cost-effective.

Perceived benefits were seen to have an insignificant effect on behavioral intention (β: −0.018; *p* = 0.717). Thus, respondents do not perceive the consistent intake of gym supplements as a preventive measure against health-related issues, which aligns with how consuming these supplements does not aid in building good health or contribute to an individual’s well-being. These findings are consistent with the study by Kiely et al. [74], which investigated the health behaviors of individuals during the pandemic. It was found that perceived benefit had no statistical significance toward a change in exercise behavior; individuals’ perceptions of the benefits of exercise did not strongly correlate with their actual exercise behavior changes. This implies that, at least in the context of the studied population during the pandemic, people were more influenced by perceived barriers than by their beliefs in the positive outcomes of exercise. Similarly, Rodrigues [58] stated that individuals consumed fitness supplements with the goal of gaining muscle, improving recovery, and enhancing performance. None have a correlation with health, as fitness supplements are not mandatory and are not endorsed to aid in the treatment of any health-related issue. These results contrast with the findings of Ghai and Sharma [75], who found that perceived benefits, alongside trust, shaped satisfaction and the intention to purchase organic food. Consumers who perceive benefits and have trust in organic foods are more likely to express a willingness to pay premium prices and experience higher satisfaction levels. This is similar with the findings of Hoseini et al. [76], in which perceived benefits significantly affected the consumption of dietary supplements, with individuals incorporating these products into their routines for various reasons. The desire for fitness, physical beauty, and skin health, alongside the intention to fill nutritional gaps and alleviate the side effects of micronutrient deficiencies, drives individuals to use supplements.

Moreover, health motivation significantly affected behavioral intention (β: 0.307; *p* < 0.001). Based on the constructs, respondents consume fitness supplements with multiple goals in mind, including increasing muscle mass, enhancing performance in fitness activities, meeting nutritional needs, and supporting the maintenance or initiation of a healthy lifestyle. They view fitness supplements as versatile tools with a range of positive impacts. The diverse reasons for consumption suggest a comprehensive approach to health, encompassing not only physical well-being but also performance and lifestyle considerations. Additionally, they see fitness supplements as instrumental in achieving various health-related objectives, from muscle building and performance enhancement to nutritional support and the promotion of a sustainable healthy lifestyle. This is aligned with the results of Sirico [46], which found that the enhancement of sport performance and overall health and wellness are primary motivations for supplement use. Additionally, Kovács’ [44] study discovered that health preservation and physical well-being were the primary reasons for taking food supplements due to individual motivations, psychological factors, and the recognition of the interconnectedness between nutrition and overall well-being.

Tehrani [77] found that diabetes patients with a history of CAM use demonstrated fewer barriers to engaging in diabetes self-care behaviors. Therefore, mitigating perceived barriers increases the probability of an individual to engage in certain behavior. In relation to the study, perceived barriers were seen to have an insignificant impact on behavioral intention (β: −0.018; *p* = 0.717). The consumers do not express challenges related to acquiring and consuming fitness supplements, specifically in terms of distance, cost, limited availability, lack of motivation, and embarrassment as barriers. This indicates that consumers have easy access to affordable fitness supplements, either due to the proximity of stores, reasonable pricing, or the availability of cost-effective alternatives. Additionally, they feel confident and motivated in their health choices, leading to a more open and positive attitude toward supplement use. This result is consistent with the study by Febian et al. [78], which found that individuals are inclined to consume functional food if they anticipate fewer barriers, such as difficult preparation and an unpalatable scent.

Self-efficacy was seen to have a significant impact on automatic (β: 0.717; *p* < 0.001) and controlled precursors (β: 0.705; *p* < 0.001). Fitness supplements aid in their confidence in different aspects of exercise and achieving their fitness goals. They contribute positively, which leads to greater adherence to routines, increased motivation, and overall fitness outcomes. This is consistent with the findings of Zhao et al. [79], which were that self-efficacy influences exercise behavior by boosting confidence in athletic abilities. Higher exercise self-efficacy contributes to a more enjoyable and enduring engagement in physical activity, fostering a lifelong habit of sports. This has also verified the results of Gacek [56], which revealed that individuals with higher self-efficacy engaged in behaviors that promote health and enhance physical fitness, and they engaged less in behaviors that are detrimental to their health, such as drinking alcoholic beverages.

The results revealed that controlled precursors have a significant effect toward behavioral intention (β: 0.272; *p* < 0.001), which based on the indicators, were voluntary evaluation of the benefits of the supplements, matching their consumption with proper diet and exercise, recognizing their usefulness, and acknowledging the value of a healthy lifestyle affect consumers’ intent to engage in activities that are aligned with fitness. This implies that controlled precursors prompt their intention to consume supplements suggesting that the precursors outweigh the impact of opposing processes that promote minimal effort [28]. Moreover, controlled precursors are classified as advanced cognitive functions, including reflective reasoning, that play a significant role in the decision-making process [80]. This indicates that respondents evaluate activities based on the outcomes that come with performing the activity. This is evident in the study by Jagim et al. [81], which found that individuals consume pre-workout, which generally contained beta-alanine, caffeine, and creatine, to make workouts easier and improve their performance.

Automatic precursors were observed to have an insignificant effect on behavioral intention (β: 0.107; *p* = 0.206). These results suggest that the involuntary cues have little influence over decision-making, indicating that respondents rely on conscious evaluation and deliberation when considering whether to consume fitness supplements. They are more likely to weigh the pros and cons and consider their goals for their consumption [82]. These results are inconsistent with the studies by Rodrigues [58], which found that past behaviors and autonomous motivation fostered by satisfaction and enjoyment were seen to be significant predictors of future intentions to exercise. This suggests that habitual actions influenced by past experiences may overshadow intentions in predicting future behavior. The insignificance of automatic precursors is consistent with the nature of this study because the decision to consume supplements is optional and heavily relies on the conscious evaluation aligned with an individual’s personal goals and nutritional needs. Based on the indicators, the findings depicted how habits and satisfaction from past behaviors are overshadowed by conscious considerations and external influences.

Lastly, with behavioral intention as the mediating factor, it was seen to have a significant role in actual consumption (β: 0.747; *p* < 0.001); respondents express the willingness to continue purchasing fitness supplements and plan to use them to improve their health in the coming periods. Additionally, they plan to pay attention to the supplements they will consume and recommend to others. This indicates a positive perception of supplements, which is a probable basis for the intention to continuously consume them for improving their fitness and endorsing others to consume them, as well. This is consistent with the results of Nystrand and Olsen [64], which were that consumers’ behavioral intention to consume functional foods was shaped by factors such as attitude, self-efficacy, and subjective norms. This suggests that individuals are motivated to consume certain products, as long as they perceive themselves as capable of doing so, and that social pressure from individuals of importance plays a pivotal role, as well. Moreover, behavioral intention toward food supplement safety was shaped by health motivation and attitude in the study by Bayır [83], indicating that positive attitude and concerns about personal health leads to the consumption of supplements, thereby predicting that supplement use will increase in the future.

## 6. Conclusions and Implications

The study contributes to existing theories, such as the Theory of Planned Behavior (TPB), Health Belief Motivation (HBM), and Theory of Effort Minimization in Physical Activity (TEMPA), by providing empirical evidence of the factors influencing consumers’ intention to consume fitness supplements. This expands the understanding of how psychological factors shape consumer behavior. The study highlights the importance of health motivation as the central driver of behavioral intention, suggesting that health-related goals play a significant role in shaping consumer decisions regarding supplement usage. Additionally, the study underscores the complexity of consumer decision-making processes, demonstrating the interplay between cognitive evaluation, self-efficacy, and automatic precursors in influencing behavioral intention. Through theoretical constructs discussed, the study provides a foundation for further research into the underlying mechanisms driving consumer behavior in the fitness supplement market, ultimately advancing the theoretical understanding of consumer decision-making.

The findings of the study can be significant for the fitness supplement industry. Firstly, companies should prioritize marketing efforts that highlight the benefits and ease of use of their products, emphasizing factors such as effectiveness, convenience, and affordability to appeal to consumers. Secondly, understanding the strong influence of health motivation, companies can tailor their product offerings and marketing to align with consumers’ health goals and aspirations, thus enhancing the perceived value of their supplements. Thirdly, educational interventions are crucial to address any misconceptions regarding supplement usage, providing evidence-based information to build consumer trust and confidence. Additionally, companies can invest in strategies to enhance consumer self-efficacy, such as providing resources and support to help consumers make informed decisions about supplement usage. Overall, by focusing on these managerial implications, companies can better meet the needs and preferences of consumers, driving sales and fostering long-term brand loyalty in the competitive fitness supplement market. Future research may opt to consider more respondents as gaining more diverse demographic context, insights, and perception, which may lead to other findings.

## Figures and Tables

**Table 1:** Demographic profile.

Factor	Characteristics	N	%
sex	male	144	57.6
	female	106	42.4
age	15–24 years old	187	74.8
	25–34 years old	51	20.4
	35–44 years old	9	3.6
	45–54 years old	2	0.8
	55–64 years old	1	0.4
	64 years old and older	0	0.0
education attainment	elementary	0	0.0
	junior high school	4	1.6
	senior high school	138	55.2
	vocational	7	2.8
	college	99	39.6
	master’s degree	2	0.8
	Ph.D.	0	0.0
employment status	student	175	70.0
	unemployed	6	2.4
	employed/self-employed	69	27.6
monthly income/allowance	less than 15,000 Php	156	58.4
	15,000–30,000 Php	52	20.8
	30,001–45,000 Php	22	8.8
	45,001–60,000 Php	16	6.0
	more than 60,000 Php	15	6.0
period of consumption of fitness supplements	less than 1 year	140	56.0
	1–2 years	72	28.8
	2–3 years	19	7.6
	more than 3 years	19	7.6
places of purchase	pharmacies	26	10.4
	fitness centers	14	5.6
	supplement stores	69	27.6
	internet/online shopping	138	55.2
	Other	3	1.2
price	less than 1000 Php	99	39.6
	1001–2000 Php	101	40.4
	greater than 2000 Php	50	20

**Table 2:** Lower-order construct validity and reliability.

Construct	Items	Mean	StD	Initial IL	Final FL	CA	CR	AVE
attitude (A)	A1	4.2	0.8	0.8	0.8	0.8	0.9	0.6
	A2	3.6	1.1	0.8	0.8			
	A3	3.9	1.0	0.8	0.8			
subjective norms (SN)	SN1	3.3	1.2	0.5	-	-	-	-
	SN2	3.1	1.2	0.6	-			
	SN3	3.8	1.0	0.8	-			
	SN4	3.7	1.1	0.8	-			
perceived behavioral control (PBC)	PBC1	4.5	0.8	0.7	0.7	0.8	0.9	0.6
	PBC2	4.3	0.9	0.8	0.8			
	PBC3	4.2	0.9	0.8	0.8			
	PBC4	4.3	0.9	0.8	0.8			
perceived effectiveness (PE)	PE1	4.4	0.7	0.8	-	-	-	-
	PE2	3.2	1.2	0.6	-			
	PE3	3.4	1.1	0.7	-			
	PE4	3.7	1.1	0.7	-			
perceived benefits (PBE)	PBE1	3.3	1.1	0.7	-			
	PBE2	3.8	1.0	0.8	-	-	-	-
	PBE3	3.8	1.0	0.8	-			
	PBE4	4.2	0.9	0.8	-			
perceived barriers (PBA)	PBA1	2.8	1.3	0.5	-	-	-	-
	PBA2	3.7	1.1	0.2	-			
	PBA3	3.0	1.4	0.5	-			
	PBA4	2.4	1.3	0.9	-	-	-	-
	PBA5	2.0	1.2	0.9	-			
health motivation (HM)	HM1	3.8	1.0	0.7	-	0.8	0.9	0.7
	HM2	4.1	1.1	0.7	-	-	-	-
	HM3	4.2	0.9	0.8	0.8			
	HM4	4.2	0.9	0.8	0.8	-	-	-
	HM5	4.2	0.9	0.8	0.8			
self-efficacy (SE)	SE1	4.2	0.9	0.9	0.9	0.9	0.9	0.6
	SE2	4.2	0.9	0.8	0.8	-	-	-
	SE3	4.2	0.9	0.8	0.8	-	-	-
	SE4	4.2	0.9	0.8	0.8	-	-	-
	SE5	4.1	0.9	0.7	0.7			
automatic precursors (AP)	AP1	4.2	0.8	0.9	0.9	0.8	0.9	0.7
	AP2	4.0	1.0	0.8	0.8	-	-	-
	AP3	4.0	0.9	0.9	0.9			
controlled precursors (CP)	CP1	4.4	0.8	0.7	0.7	0.8	0.9	0.6
	CP2	4.4	0.8	0.8	0.8	-	-	-
	CP3	4.3	0.8	0.8	0.8	-	-	-
	CP4	4.1	0.9	0.8	0.8	-	-	-
	CP5	4.6	0.7	0.7	0.7			
behavioral intention (BI)	BI1	3.7	1.2	0.8	0.8	0.8	0.9	0.6
	BI2	4.0	1.0	0.8	0.8	-	-	-
	BI3	4.3	0.8	0.7	0.7	-	-	-
	BI4	4.1	0.9	0.8	0.8			
actual consumption (AC)	AC1	4.2	0.9	0.8	0.8	0.8	0.9	0.7
	AC2	4.1	0.9	0.8	0.8	-	-	-
	AC3	4.1	0.9	0.8	0.8	-	-	-
	AC4	4.2	0.8	0.8	0.8			

Note: StD—standard deviation; AVE—average variance extracted; CA—Chronbach’s alpha; CR—composite reliability; IL—initial loading; FL—final loading; A—attitude; SN—subjective norms; PBC—perceived behavioral control; PE—perceived effectiveness; PBE—perceived benefits; PBA—perceived barriers; HM—health motivation; SE—self-efficacy; AP—automatic precursors; CP—controlled precursors; BI—behavioral intention; AC—actual consumption.

**Table 3:** Discriminant validity.

Fornell–Lacker Criterion								
	AP	AC	A	BI	CP	HM	PBC	SE
AP	0.846	-	-	-	-	-	-	-
AC	0.691	0.829	-	-	-	-	-	-
A	0.591	0.614	0.795	-	-	-	-	-
BI	0.634	0.747	0.66	0.777	-	-	-	-
CP	0.666	0.741	0.587	0.682	0.762	-	-	-
HM	0.615	0.738	0.583	0.669	0.647	0.825	-	-
PBC	0.533	0.565	0.595	0.565	0.563	0.536	0.784	-
SE	0.717	0.727	0.656	0.73	0.705	0.676	0.597	0.799
Heterotrait-Monotrait Ratio								
	AP	AC	A	BI	CP	HM	PBC	SE
AP	-	-	-	-	-	-	-	-
AC	0.837	-	-	-	-	-	-	-
A	0.72	0.722	-	-	-	-	-	-
BI	0.804	0.812	0.809	-	-	-	-	-
CP	0.804	0.819	0.68	0.836	-	-	-	-
HM	0.776	0.811	0.709	0.843	0.795	-	-	-
PBC	0.643	0.662	0.687	0.697	0.684	0.655	-	-
SE	0.844	0.848	0.767	0.771	0.822	0.82	0.697	-

**Table 4:** Model fit indices.

Parameters	Estimates	Suggested Cutoff	Reference
SRMR	0.068	<0.08	Hu and Bentler [66]
Chi-Square	3.400	<5.00	Hooper et al. [67]
NFI	0.921	>0.90	Baumgartner and Homburg [68]

**Table 5:** Hypothesis results.

Hypothesis	Relationship	β-Values	ρ-Values	Decision
1	attitude → behavioral intention	0.257	<0.001	accept
2	subjective norms → behavioral intention	0.021	0.688	reject
3	perceived behavioral control → behavioral intention	0.193	0.004	accept
4	percevied effectiveness → behavioral intention	−0.057	0.429	reject
5	health motivation → behavioral intention	0.307	<0.001	accept
6	perceived benefits → behavioral intention	0.087	0.214	reject
7	perceived barriers → behavioral intention	−0.018	0.717	reject
8	self-efficacy → automatic precursors	0.717	<0.001	accept
9	self-efficay → controlled precursors	0.705	<0.001	accept
10	automatic precursors → behavioral intention	0.107	0.206	reject
11	controlled precursors → behavioral intention	0.272	<0.001	accept
12	behavioral intention → actual consumption	0.747	<0.001	accept

## Data Availability

The data presented in this study are available on request from the corresponding author. The data are not publicly available due to privacy restrictions.

## Appendix A

**Table A1:** Measure items.

Variable	Code	Description	References
attitude	A1	I think the money spent on fitness supplements is worthwhile	[15]
	A2	It is important to consume fitness supplements	[15]
	A3	It is valuable to consume fitness supplements	[15]
	A4	It is useful to consume fitness supplements	[15]
subjective norm	SN1	Most people I know consume fitness supplements	[30]
	SN2	Most people who are important to me would think I should take fitness supplements	[30]
	SN3	People who are important to me would approve of me consuming fitness supplements	[30]
	SN4	Most people like me consume fitness supplements	[26]
perceived behavioral control	PBC1	Consuming fitness supplements is completely under my control	[60]
	PBC2	Consuming fitness supplements makes me feel like I am taking care of my physical performance	[60]
	PBC3	Consuming fitness supplements is easy for me	[60]
	PBC4	I am able to consume fitness supplements if I want to	[60]
perceived effectiveness	PE1	I think the use of supplements is beneficial	[84]
	PE2	I think it is necessary to use supplements	[84]
	PE3	I think that fitness supplements have little to no side effects	[84]
	PE4	I think fitness supplements that maintain a high level of energy and activity during the day are necessary	[84]
perceived benefits	PBE1	Regular consumption of gym supplements prevents health-related problems	[15]
	PBE2	I believe I feel better, health wise, if I consume gym supplements	[15]
	PBE3	Regular consumption of gym supplements would help build good health	[15]
	PBE4	Consumption of gym supplements also improves the way the body feels and looks	[15]
perceived barriers	PBA1	Places for me to purchase fitness supplements I need are too far away	[85]
	PBA2	It costs too much money to purchase the fitness supplements I need	[85]
	PBA3	There are too few places for me to purchase the fitness supplements I need	[85]
	PBA4	I lack motivation to consume fitness supplements	[85]
	PBA5	I am too embarrassed to consume fitness supplements	[85]
health motivation	HM1	I consume fitness supplements to preserve my health	[44]
	HM2	I consume fitness supplements to increase my muscles mass	[44]
	HM3	I consume fitness supplements to boost my performance in fitness aspects	[44]
	HM4	I consume fitness supplements to adhere and supplement my nutritional needs	[44]
	HM5	I consume fitness supplements to aid in maintaining/beginning a healthy lifestyle	[44]
self-efficacy	SE1	Consuming fitness supplements aids in my confidence to perform resistance exercises	[86]
	SE2	Consuming fitness supplements aids in my confidence to challenge myself during workouts	[87]
	SE3	Consuming fitness supplements aids in my confidence to perform physical exercises regularly	[88]
	SE4	Consuming fitness supplements makes it easy to stick to my fitness goals	[89]
	SE5	I find it easy to make sure I meet the recommended dosage of my fitness supplements	[89]
automatic precursors	AP1	I feel good after consuming fitness supplements	[28]
	AP2	After consuming fitness supplements, I think of consuming supplements in the following days	[90]
	AP3	If I were asked about my feelings regarding consuming fitness supplements, I would say it is enjoyable	[57]
controlled precursors	CP1	I evaluate the benefits and outcomes of fitness supplements before consuming it	[57]
	CP2	I think matching my consumption with proper diet and exercise may take a little effort but is beneficial for me	[28]
	CP3	I think exerting effort to consume fitness supplements is worthwhile	[91]
	CP4	Regular consumption of supplements will enable me to become physically and mentally healthy	[28]
	CP5	I think having a healthy lifestyle is rewarding in the long run	[92]
behavioral intention	BI1	I intend to purchase fitness supplements during the next weeks	[93]
	BI3	I plan to use fitness supplements for my health in the coming weeks.	[94]
	BI4	I plan to pay attention to the fitness supplements I consume.	[94]
	BI5	I would recommend fitness supplements to others.	[95]
actual consumption	AC1	My consumption of fitness supplements has improved my physical health	[96]
	AC2	My consumption of fitness supplements gives me peace of mind about my physical health	[96]
	AC3	My consumption of fitness supplements has given me more control of my physical health	[96]
	AC4	I would continuously consume fitness supplements for my fitness journey.	[96]
